# Full-Field Strain Measurement and Numerical Analysis of a Microalloyed Steel Subjected to Deformation with Strain Path Change

**DOI:** 10.3390/ma13235543

**Published:** 2020-12-04

**Authors:** Paulina Lisiecka-Graca, Janusz Majta, Krzysztof Muszka

**Affiliations:** Faculty of Metals Engineering and Industrial Computer Science, AGH University of Science and Technology, 30 Mickiewicza Ave., 30-059 Krakow, Poland; majta@metal.agh.edu.pl (J.M.); muszka@agh.edu.pl (K.M.)

**Keywords:** work hardening model, strain path changes, DIC analysis, dislocation density

## Abstract

This study presents an effective technique for taking advantage of the full-field measurement method of Digital Image Correlation (DIC) for the assessment of the strain distribution during the metal forming process when the strain path change was performed. The applied methodology is based on the combination of a numerical simulation for the stress calculation and full-field surface strain measurement in a forward/reverse three-point bending test. In the numerical part, the Chaboche model and dislocation density-based model were selected and verified in terms of the prediction of a softening/hardening effect occurring during strain reversal. The Chaboche model parameters identification procedure, on the basis of a cyclic torsion test, combined with inverse analysis, was also described. The results of the study showed the advantages and disadvantages of both of the analyzed work hardening models. The obtained results were analyzed in the light of the deformation inhomogeneity and reorganization of the dislocation structure during the cyclic deformation test.

## 1. Introduction

During metal forming processes, the material undergoes a complex path of deformation that often involves not only the high level of deformation, but also microstructural inhomogeneity, which makes the prediction of material behavior especially difficult [1,2,3]. One of the most important phenomena in such processes is the Bauschinger effect, which is a measure of the resistance of a material to strain reversal [4,5,6,7]. The understanding of the Bauschinger effect is especially important in the case of cold and warm metal forming, when recovery processes are limited. The Bauschinger effect is characterized by a significant decrease of the resistance to flow stress after the change of deformation direction, and can be accompanied by an apparent difference between the monotonic and reversed flow behaviors at large accumulated strains [8]. In light of this, the constitutive model should be able to predict the softening/hardening effects, as such effects can significantly influence the strain distribution, and may lead to flow localization. The effect of strain path as a key processing parameter has been studied recently [9,10,11]. The main aim of the present paper is to discuss modelling possibilities of the cyclic deformation behavior of microalloyed steel, based on the two different models with respect to their strain path sensitivity. Two-dimensional Digital Image Correlation (DIC) analysis was applied as a verification tool for the numerical simulation. The DIC system is employed to measure the deformation of specimens affected by the cyclic strain path changes during the plastometric tests. In order to determine the local accumulation of the deformation energy, the measurements of displacements and the resulting strains were used in the analysis of the mechanical properties assessment. The combination of these two methods (DIC analysis for the strain distribution measurement and numerical simulation for the flow stress calculation) allows better prediction of the most important phenomena that occur during the complex history of deformation.

## 2. Experiment Procedure

The effects of the strain path changes on the material behavior were investigated using a forward/reverse three-point bending test. The specimens used in the tests had a gauge length of 46 mm, width of 5.5 mm and thickness of 4.4 mm. The experiments were performed at room temperature with the displacement rate of 1 mm/min. The experimental procedure was combined with DIC analysis. During the tests, the flexure load and flexure extension were recorded. The obtained data were converted into equivalent plastic stress vs. equivalent plastic strain curves using the standard equations. These curves were used in the modelling part.

### 2.1. Material

**Figure 1:**
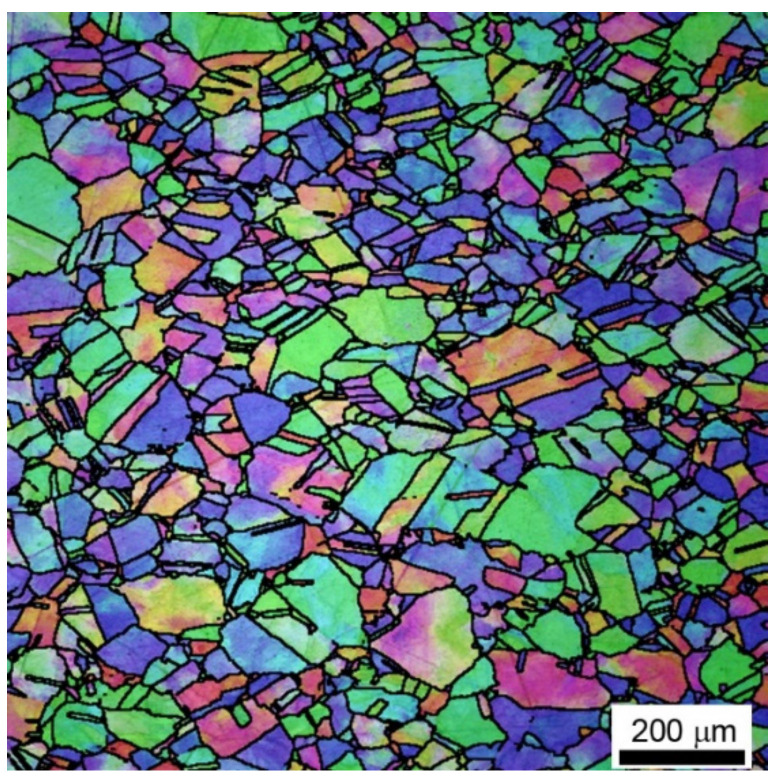
Initial microstructure of the microalloyed austenite.

The investigated material was the hot-rolled microalloyed austenite (model alloy) with basic chemical composition presented in Table 1. The major advantage of the studied model alloy is the fact that austenite phase is stable at room temperature. In the investigated material, a micro-alloying addition of Nb was added to cause a precipitation strengthening mechanism, which plays a significant role in the formation of the dislocation substructure [12,13,14,15]. The initial microstructure of the investigated material is presented in Figure 1.

### 2.2. Digital Imaging Strain Analysis Method

In the current work, the DIC system was employed to measure the strain distribution and propagation during the cyclic three-point bending test. The evaluations of the strain localization behavior and the strain distribution in the tests were performed using Istra4D software (version 4.4.1.354). In the present study, a two-dimensional DIC technique was applied. In the DIC method, a stochastic pattern is painted on the sample to determine the displacement of the objects on the sample using the image correlation calculation [16,17,18,19,20]. The system of two high-resolution CCD cameras (Allied Vision, Stadtroda, Germany) enables the tracking of the position of the same physical pattern shown in the reference and deformed images. This procedure allows the determination of the object deformation in a plane parallel to the image plane of the camera. If the analyzed object is observed by two cameras at different angles, the position of each object point is focused on a specific pixel in the camera plane. The three-dimensional coordinates of any surface point in the space can only be calculated if the information about both cameras (position of both cameras relative to each other, and magnifications of the lenses) and all image parameters are known. In the present work, the system of two cameras with lenses of 1.9/35 was used. For the calibration of the system, the calibration target of 9 × 9 with a total side length of 30 mm was utilized.

**Figure 2:**
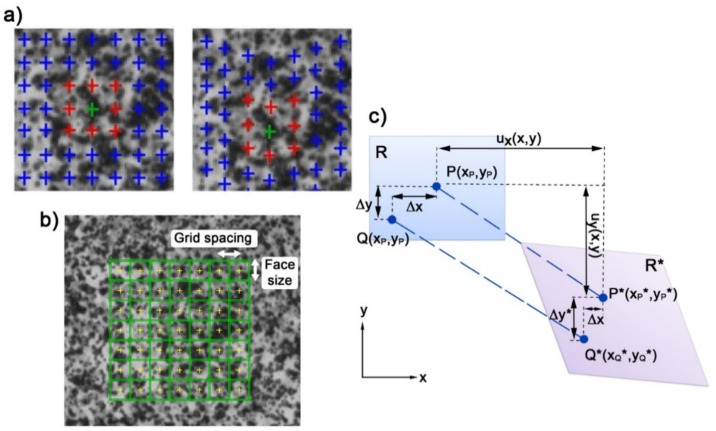
(**a**) Recognition of displacement based on an analysis of pixels in the area of facets for the references and deformed image; (**b**) face size and grid specking using the measurements of the samples; and (**c**) subset before and after deformation.

The important aspects are that the object surface shows a clear structure of the speckles, in order to allow the algorithms to correlate identical points from both cameras [21]. Speckle pattern quality (size of the speckles and contrast), as well as the size of the facets, have a strong influence on the accuracy and resolution of measurements (Figure 2a,b). The described correlation parameters were used in the present study. The face size was equal to 19 px, and the grid spacing to 17 px. The maximum value of accuracy used in the present work was 0.1 px.

The basic processing work is done by Istra4D, which calculates the average grey scale intensity over the subset in the reference image and the image recorded after deformation, and compares them. The algorithm is based on the tracking of the grey value pattern in small local neighbourhoods (Figure 2c). 2–D or 3–D displacements *u*(*x*) and displacement derivatives are estimated by matching (correlating) the image texture, i.e., light intensity (grey level) distributions, which are *f*(*x*) and *g*(*x*), respectively. In the DIC method, optical flow conservation is required (Equation (1)):

(1) $$g\left(x\right)=f\left(x\right)+u\left(x\right)$$

Selecting a single subset from the reference image, the image after deformation is searched for the same grey level distribution [22]. In the DIC methods, the important components are the shape functions. The shape function is a component to relate the coordinates of the pixel of the interest in the reference and deformed image, as well as the target variables to determine (Figure 2). Points *P* and *Q* move to be points P^*^ and Q^*^ under the load (Figure 2c). If the displacement component of point $P\left(x,y\right)$ in *x* and *y* directions are assumed to be $\left(u_{x},\;u_{y}\right)$, then the displacement component of point *Q* in *x* and *y* directions can be written by Equations (2) and (3):

(2) $$u_{\mathrm{xQ}}=u_{x}+\frac{\partial u_{x}}{\partial x}dx+\frac{1}{2}\frac{\partial^{2}u_{x}}{\partial x^{2}}dx^{2}+\frac{\partial u_{x}}{\partial y}dy+\frac{1}{2}\frac{\partial^{2}u_{x}}{\partial y^{2}}dy^{2}+\frac{\partial^{2}u_{x}}{\partial x\partial y}dxdy$$

(3) $$u_{\mathrm{yQ}}=u_{y}+\frac{\partial u_{y}}{\partial x}dx+\frac{1}{2}\frac{\partial^{2}u_{y}}{\partial x^{2}}dx^{2}+\frac{\partial u_{y}}{\partial y}dy+\frac{1}{2}\frac{\partial^{2}u_{y}}{\partial y^{2}}dy^{2}+\frac{\partial^{2}u_{y}}{\partial x\partial y}dxdy$$

Then, the coordinate of the Q* can be obtained by Equations (4) and (5):

(4) $$x_{Q}^{*}=x+dx+u_{\mathrm{xQ}}$$

(5) $$y_{Q}^{*}=y+dy+u_{\mathrm{yQ}}$$

Correlation function is another important component, which is used to calculate a coefficient which represents the similarity between two sets of data. The commonly used correlation functions can be divided into two groups: cross-correlation function; and sum of squared differences function. In the presented study, we used the cross-correlation function. The function is presented in Equation (6), and the maximum of *C*_CC_ represents a maximum similarity [23].

(6) $$C_{\mathrm{CC}}=\overset{M}{\underset{i,j=-M}{\sum }}(f(x_{i},\;y_{i})\times g\left(x_{i}^{*},\;y_{i}^{*}\right))$$

## 3. Modelling

The significant element of the computer modelling procedure is choosing the appropriate work hardening model. In the current work, the verification of the existing hardening models was performed with the help of DIC analysis. This comparison allowed one to check the correctness of the prediction of the materials’ behavior during the numerical simulation of the presented process. The main aim of the current work was a verification of the existing work model parameters under a complex strain state (using a forward/reverse three-point bending test).

As already mentioned, in the selection process of the proper work hardening model for the computer simulation of the strain path change process, an important element is to include the Bauschinger effect. In the deformation characterized by a simple strain path, it is possible to use the isotropic model where the yield surface can only change its radius. Nevertheless, in most metal forming processes, material is subjected to a more complex history of deformation. Then, there is a need to apply a complex solution which includes both a change in the radius of the yield surface and a change of its position in the stress space.

### 3.1. Chaboche Model

In the present study, the first analyzed model was the Chaboche model [24]. In this model, the isotropic and kinematic hardening parts were adequately employed. Equation (7), which describes the isotropic part of the Chaboche model, includes the size of the yield surface (*σ*^0^), maximum change in this surface (*Q*) and the change rate of this surface during the plastic deformation (*b*).

(7) $$\sigma^{0}=\sigma_{0}+Q\left(1-exp^{-b\varepsilon }\right)$$

The kinematic part of the presented approach is set out as a sum of the backstresses (α). The parameters presented in Equation (8) control the position of the stress for each backstress. The description of the kinematic components of the presented model consists of the initial kinematic hardening modulus (*C*_k_) and the rate of decreasing this modulus with respect to the increasing plastic deformation (γ_k_).

(8) $$\dot{\alpha }=\frac{C_{k}}{\sigma^{0}}\left(\sigma -\alpha \right)\varepsilon -\gamma_{k}\alpha \varepsilon$$

In order to identify the model parameters, the optimization tool utilizing inverse approach was used. The optimization was made based on another experiment: the forward/reverse torsion test [25]. The inverse method was used in order to determine the coefficients of the work hardening model given by Equations (7) and (8). Inverse analysis is an iterative process driven by an optimization algorithm, where the objective function is defined and minimized with respect to the appropriate criteria. The applied inverse analysis steps are as follows:

- Before starting the iterative process, there is a need to select the parameters to be identified and define the ranges of their possible values, delimiting the parameter search space. The input data of the process are the experimental data from the cyclic test. In the current work, the searching model parameters were $\sigma_{0},\;Q,\;b,\;C_{k},{\;\gamma }_{k}$.
- In the next step, the Finite Element method model of the considered process is run, and a direct solution is obtained (in this work, Abaqus Standard code was used). Then, the simulation results are passed to the optimization module and the objective function value is calculated. In the present study, the objective function is defined as an error between measured and calculated data by the following Equation (9):
  (9) $$\phi =\sqrt{\frac{1}{N_{\mathrm{ps}}}\overset{N_{\mathrm{ps}}}{\underset{j=1}{\sum }}{\left(\frac{F_{\mathrm{cji}}\left(x,p_{i}\right)-F_{\mathrm{mji}}}{F_{\mathrm{mji}}}\right)}^{2}}$$
  where: *N*_ps_-number of load measurements; *F*_cji_ and *F*_mji_-calculated and measured loads; *p*-vector of process parameters (strain rate, temperature); and *x*-vector of coefficients in the combined hardening rule.
- The last step is the evaluation of the obtained values of the objective function. If the solution meets the convergence criteria and the solution error is satisfactory, the obtained parameters can be considered as the optimum process parameters. Otherwise, the minimization process of the objective function, with respect to the process parameters, is performed again until the optimal solution has been found. In this work, the Simplex algorithm was used as the minimization function. The obtained model parameters for the microalloyed austenite are presented in Table 2.

### 3.2. Dislocation Density-Based Rauch, Gracio, Barlat Model (RGB Model)

Microalloyed steels are characterized by the presence of the solid solution and precipitation strengthening mechanisms. These mechanisms have a significant impact on the hardening process, mainly due to a delay in the dislocation substructure reorganization process [26]. Due to this fact, the RGB work-hardening model, which includes the dislocation density as an internal variable, was used. The dislocation substructure observed after forward deformation is significantly changed in the second (reverse) deformation stage. The proposed RGB model assumed that the dislocation density is divided into two main components related to the forward-loading and reverse-unloading of the dislocation substructure. In the present model, the general formula for the stress calculation (Equation (10)) is based on the Kocks model [27], and includes the stress associated with the lattice friction, solid solution (τ_0_) and the internal variable (*X*).

(10) $$\sigma =\sigma_{0}+M\left[X+\left(1-\alpha \right)\left(\tau -\tau_{0}\right)\right]$$

The mentioned internal variable *X* describes the rapid changes of the stress under reverse straining, and includes a constant, which characterizes the dynamic changes of the backstress (*C*_x_).

(11) $$\dot{X}=C_{x}\left(X_{s}\dot{\gamma }-X\left|\dot{\gamma }\right|\right)$$

The parameter *X*_s_ is described as follows: $X_{s}=\alpha (\tau -\tau_{0})$. For the shear stress calculation, it is necessary to know the forward ($\rho_{f}$) and reverse ($\rho_{r}$) dislocation density components and the factors which describe the dislocation interactions (α). The shear stress is described by the following equation:

(12) $$\tau =\tau_{0}+\alpha Gb\sqrt{\rho_{f}+\rho_{r}}$$

The components of the dislocation density parameters were calculated using the following equations:

(13) $$\frac{d\rho_{f}}{d\varepsilon }=\frac{1}{b\Lambda }-f\rho_{f},\;\frac{d\rho_{r}}{d\varepsilon }=\frac{1}{b\Lambda }\cdot \frac{\rho_{r}}{{\dot{\rho }}_{f}}$$

(14) $$\rho_{f}\left(\gamma_{f}=0\right)=\left(1-p\right){\dot{\rho }}_{f},\;\rho_{r}\left(\gamma_{f}=0\right)=p{\dot{\rho }}_{f}$$

In Equations (13) and (14), the storage of a thermal part depends on the mean free path Λ of the mobile dislocations. The factor *f* describes the thermally activated recovery that depends on the temperature and strain rate. The model parameters used for the calculations have been taken from the literature. The initial dislocation density was estimated using the Holt’s model [28]. The major assumption of this model is the occurrence of the critical distance, at which dislocation interactions can be neglected. Then, based on the model, the dislocation cells’ size can be calculated. The detail description of the presented models can be found in [29].

## 4. Discussion

**Figure 3:**
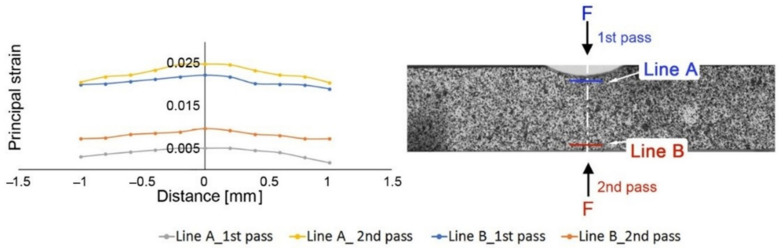
Strain distribution along the line A and B for the microalloyed austenite obtained in the Digital Image Correlation (DIC) analysis.

In the presented work, the DIC strain measurement was applied to study the strain localization behavior under a forward/reverse three-point bending test. During the cyclic test, the strain accumulation resulting from previous deformation (prestraining) can be observed. Figure 3 shows the differences in principal strain distribution during the process for the investigated material obtained from the DIC analysis. The maximum strain value of the first and second passes were represented as blue and yellow lines, respectively. The maximum value of the strain distribution for the microalloyed austenite in the first pass has a lower value than in the second pass. Furthermore, the strain accumulation after the first pass was clearly visible in the microalloyed steel.

**Figure 4:**
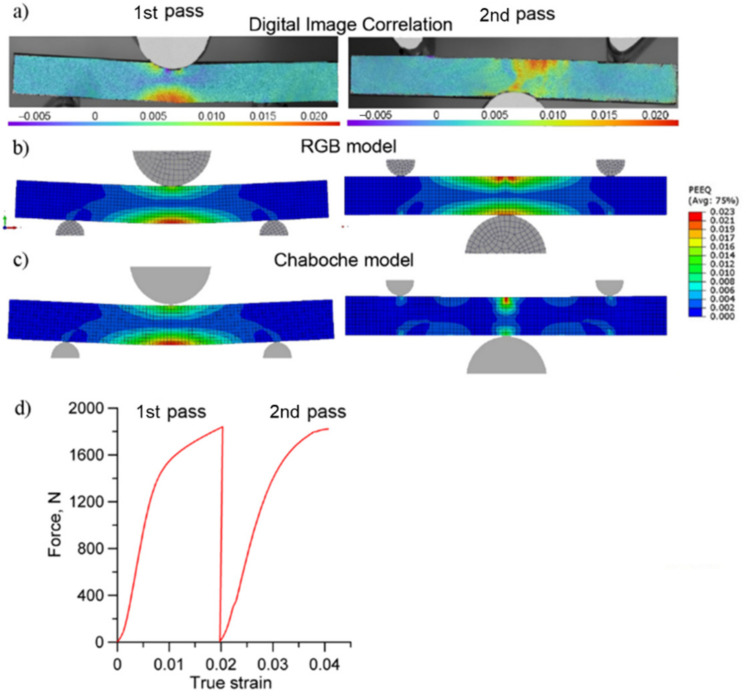
(**a**) Principal strain distribution maps obtained from the DIC analysis; (**b**) the numerical simulation with RGB model; and (**c**) the Chaboche model; as well as (**d**) the force-strain curve obtained from the experiment and DIC analysis.

An experimental curve was created based on the strain taken directly from the DIC measurement and stress calculated based on the load from the testing machine (Figure 4d). The precision of the DIC method allowed for high accuracy in the strain concentration analysis, and was proved to be a good validation for the numerical modelling.

**Figure 5:**
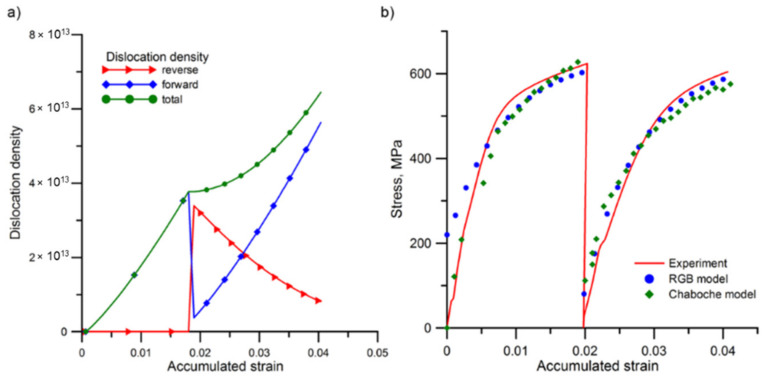
The components of the dislocation density which were calculated based on: (**a**) the RGB model; and (**b**) the stress-strain curve obtained in the numerical simulation and experiment for the forward/reverse three-point bending test.

The results obtained using the DIC technique were compared with data from the numerical simulation. In the computer modelling, the principal strain distribution, taken from the simulation when the Chaboche model was used, is more concentrated in the second pass (taking up about 1 mm of the total sample length) than in the DIC analysis and the RGB model (where it took about 4 mm of total sample length) (Figure 4). The value of the principal strain obtained from the Chaboche and RGB models was similar, and equal to 0.023. Nevertheless, the numerical simulation results for the RGB and Chaboche models captured corrected both the value and concentration of the principal strain. The results obtained from the RGB model based on the model parameters from the literature only show similar strain distribution to the Chaboche model in the first pass. However, the presented approach in the RGB model shows a better ability to track the essential features observed in the investigated material, and provide the possibility to predict the material behavior under strain path change more accurately than the Chaboche model. As already mentioned, in this model the stress is correlated by two dislocation density components that are related to the direction of the deformation. An additional advantage of the presented approach is its capability of tracking the evolution of dislocation density during the process, which is presented in Figure 5a. The change in the rate of the increase of total dislocation density, especially after reversing the direction of deformation, can be connected with the rapid disappearance of a significant amount of dislocations created in the first pass, as a result of the applied strain path change.

A comparison of the differences between calculated and measured flow stress curve using both models is presented in Figure 5b. The flow behavior and flow stress softening resulting from strain reversal were included in both models. In the Chaboche model, the underestimation of the stress level can be observed, while in the case of the microalloyed austenite it can be due to the lack of the explicate representation of the precipitation hardening in the model. It can be speculated that in this particular case, the Chaboche model is not sufficient to take into account the effect resulting from complex strengthening mechanisms that operate in this material. Nevertheless, the qualitative comparison of room temperature flow curves shows a good trend, as well as both models’ capability in the prediction of hardening effects.

## 5. Conclusions

DIC provides full-field strain measurements well into the strain accumulation region. This method allows one to measure the true strain at any point in the specimen, while the average strain measured by the extensometer can be insufficient. Both of the selected work hardening models show good trends and model capability in the prediction of hardening effects. Nevertheless, the solutions based on the dislocation density-based model gave better convergence than the Chaboche model. Based upon the results from the presented work, the following conclusions can be drawn:

- In the first stage of the second pass of the deformation, the rate of increase in total dislocation density decreased, which can be involved with the rapid disappearance of a significant amount of dislocations that were stored during the strain reversal.
- Applying the DIC technique to the strain distribution measurements has allowed the assessment of the residual strain accumulation resulting from first pass of deformation after changes of the deformation direction. This is impossible using traditional methods, where only the average strain during the process can be measured.
- The Chaboche model parameters identified based on the cyclic torsion tests can be successfully used for the simulation of other processes where the strain path change occurred. The quantitative assessment of these parameters cannot be overestimated from the point of view of the proper estimation of the deformation effects, especially work hardening.

The discussed research results allowed the demonstration of the key role of the correct selection of the work hardening mechanism in computer simulations of deformation processes characterized by a complex strain path. The appropriate mapping of the dislocation substructure formation process and the appropriate sensitivity of the work hardening model to a change in the loading direction makes it possible to use a computer simulation to design and control various straining with complex deformation modes. It was also shown how important it is to correctly identify the parameters of the applied model in the conditions of cyclic deformation, and the DIC system was used for experimental verification. Calibration measurements of the applied model were made in relation to the deformation processes with a known change of the strain path.

## Figures and Tables

**Table 1:** Basic chemical composition (wt.%).

Material	C	Mn	Si	Ni	Co	N	Nb
**Microalloyed Austenite**	0.047	1.64	0.30	30.8	0.022	0.0042	0.037

**Table 2:** Chaboche model parameters identified for the forward/reverse torsion test.

Material Parameter	$\sigma_{0}$	Q	b	$C_{k}$	$\gamma_{k}$
**Values of the Parameters**	58.94	−0.42	0.02	136,850	111.15
